# Protective Immune Responses to Dengue Virus Infection and Vaccines: Perspectives from the Field to the Bench

**DOI:** 10.3389/fimmu.2015.00075

**Published:** 2015-02-18

**Authors:** Scott B. Halstead, Simona Zompi

**Affiliations:** ^1^International Vaccine Institution, Seoul, South Korea; ^2^Division of Experimental Medicine, University of California San Francisco, San Francisco, CA, USA; ^3^Immunology and Global Health Consulting, Oakland, CA, USA

**Keywords:** dengue, antibodies, T cells, myeloid cells, NK cells, immunity, protection, vaccines

Dengue research is in turmoil following confusing efficacy reports from large-scale phase III clinical studies on the lead candidate tetravalent dengue vaccine (1–3). Within the context of the current understanding of immunity in dengue or immunity to other vaccines, the observed failures of protection cannot be adequately explained. These results comprise the background to this review of contemporary research on protective immunity in dengue, summarized by Slifka (4). Studies on wild-type dengue virus (DENV) infections of humans since World War II have revealed a consistent pattern of cross-protection after a single DENV infection against infection with a different DENV. Inapparent infections or mild disease accompany sequential DENV infections spaced at relatively short intervals (<1.4–1.9 years), while overt and severe disease accompany sequential infections at longer intervals. Grange et al. provide an analytical review of inapparent DENV infections published so far in the literature (5). It has been asked whether these inapparent infections serve as a major reservoir for the sustained infection of *Aedes aegypti*. Parameters of infection of *A. aegypti* by feeding on humans with dengue illnesses described here by Carrington et al. are a model for research directed at answering this question (6). An overview report by Endy (7) on the spectrum of human responses to wild-type DENV infection, from inapparent to hospitalized severe dengue, provides evidence that heterotypic DENV plaque-reduction neutralizing antibodies do not predict protection against a second DENV. This was the central feature of the Sanofi tetravalent dengue vaccine trial in Thai children (1). DENV 2 neutralizing antibodies uniformly were raised by three doses of vaccine yet failed to protect against symptomatic DENV 2 infections (1).

A longitudinal study on human immune responses to wild-type DENV infection describes how heterotypic immunity modulates disease, including evidence that cellular immunity contributes to protection (8). Weiskopf and Sette show that CD8+ T cells contribute to protection against disease with second DENV infections by targeting epitopes on non-structural antigens (8). In the Sanofi tetravalent chimeric vaccine, this T cell contribution may be missing as DENV non-structural proteins are not present in the vaccine, replaced by those of yellow fever (1–3). Studies on humans and animal models, summarized by Petitdemange et al. (9), find that antibody-dependent cell-mediated cytotoxicity (ADCC) and natural killer (NK) cells contribute to controlling early-stage viral infections. Since most human DENV infections are silent, NK cell-mediated protection may dominate (9, 10). This possibility is illustrated by observations from Cuba and Vietnam, reviewed by Beltran and Lopez-Verges (10), where differential distribution of alleles of the MHC-Class I chain-related genes A or B (MICA or MICB) suggest that NK responses have been suppressed in those individuals who developed severe disease. Cells of the immune system, including dendritic cells (DCs), monocytes (Mo), and macrophages (Mφ) serve as hosts of DENV infection. Immature DCs express DC-SIGN, a universal receptor for DENV. Immature DCs evolve from blood Mo that have migrated into the skin. In a mouse model, Schmid et al. show that immature DCs are initial sites of infection and once infected become mature and migrate to regional lymph nodes (11). Mature DCs lose DC-SIGN but gain Fc receptors (FcRs) and can be infected efficiently by infectious immune complexes. Different FcRs on Mo and Mφ interact with specific isotypes of IgG. When infectious DENV immune complexes attach to Mo and Mφ FcγRIIA a signal is sent suppressing interferon (IFN) type I production leading to the enhanced virus production (11).

A broad range of subhuman primate species are readily infected with wild-type or attenuated DENV. But, monkeys do not respond to infection with a disease mimicking the dengue vascular permeability syndrome (DVPS). Nonetheless, immune responses and protection to challenge in monkeys are closely similar to those observed in humans. Sariol and White review the utility and limitations of this animal model (12). Monkeys inoculated with tetravalent Sanofi and Takeda live-attenuated chimeric vaccines revealed the same dominance of DENV 4 and DENV 2-driven immune responses and protection observed in humans, respectively. T cell immune responses are scarcely studied in monkeys. In contrast, in mouse models, as shown by Zellweger and Shresta (13), adoptive transfer of T cells demonstrate the important contribution of the T cell component to protection following a first or second DENV infection. Mice lacking receptors to type I IFN, however, do have a pathophysiological response closely similar to DVPS. In these mice, suboptimal doses of DENV2 result in mild illness. In the presence of enhancing concentrations of dengue antibodies, i.e., sub-neutralizing concentrations that induce antibody-dependent enhancement or ADE, the same dose of DENV induces lethal disease (13).

The review is completed by a description by Ambuel et al. (14) of the successful immunization of cynomolgous monkeys using two doses of a DENV2 chimeric tetravalent vaccine given at day 0 (rapid immunization strategy or RIS), as compared to the traditional prime and boost given 2 months later. As evidence of solid protection, when challenged with DENV 2, animals were protected against viremia with no boost in DENV 2 neutralizing antibodies, showing that the RIS induced a sterilizing immunity (14). Another important feature of this trial was the demonstration of T cell immunity to DENV 2 non-structural proteins with collateral cross-reactive T cell immunity to other DENV types (14). This original article shows that RIS could be very useful in endemic areas to increase compliance to vaccination schedules and reinforce the necessity to study, in more detail, protective dengue-induced T cell immunity.

## Conflict of Interest Statement

The authors declare that the research was conducted in the absence of any commercial or financial relationships that could be construed as a potential conflict of interest.
